# Use of Cell and Genome Modification Technologies to Generate Improved “Off-the-Shelf” CAR T and CAR NK Cells

**DOI:** 10.3389/fimmu.2020.01965

**Published:** 2020-08-07

**Authors:** Michael A. Morgan, Hildegard Büning, Martin Sauer, Axel Schambach

**Affiliations:** ^1^Institute of Experimental Hematology, Hannover Medical School, Hanover, Germany; ^2^REBIRTH Research Center for Translational Regenerative Medicine, Hannover Medical School, Hanover, Germany; ^3^Department of Pediatric Hematology, Oncology, and Blood Stem Cell Transplantation, Hannover Medical School, Hanover, Germany; ^4^Division of Hematology/Oncology, Boston Children’s Hospital, Harvard Medical School, Boston, MA, United States

**Keywords:** chimeric antigen receptor, T cell, immunotherapy, genome editing, CRISPR-Cas9

## Abstract

The broad success of adoptive immunotherapy to treat human cancer has resulted in a paradigm shift in modern medicine. Modification of autologous and allogenic immune cells with chimeric antigen receptors (CAR) designed to target specific antigens on tumor cells has led to production of CAR T and CAR NK cell therapies, which are ever more commonly introduced into cancer patient treatment protocols. While allogenic T cells may offer advantages such as improved anti-tumor activity, they also carry the risk of adverse reactions like graft-versus-host disease. This risk can be mitigated by use of autologous immune cells, however, the time needed for T and/or NK cell isolation, modification and expansion may be too long for some patients. Thus, there is an urgent need for strategies to robustly produce “off-the-shelf” CAR T and CAR NK cells, which could be used as a bridging therapy between cancer diagnosis or relapse and allogeneic transplantation. Advances in genome modification technologies have accelerated the generation of designer cell therapy products, including development of “off-the-shelf” CAR T cells for cancer immunotherapy. The feasibility and safety of such approaches is currently tested in clinical trials. This review will describe cell sources for CAR-based therapies, provide background of current genome editing techniques and the applicability of these approaches for generation of universal “off-the-shelf” CAR T and NK cell therapeutics.

## Introduction

The clinical usefulness of cellular therapies was increasingly demonstrated through decades of successful hematopoietic stem cell transplantations (HSCT) in both autologous and allogeneic settings. In the case of allogeneic HSCT, some patients develop a complication called graft-versus-host disease (GVHD) due to cytotoxic alloreactivity of donor T cells that were transferred from the donor graft and which destroy tissues in the recipient. GVHD occurs due to immuno-incompatibility, e.g., human leukocyte antigen (HLA) mismatches between the donor and recipient. Although GVHD can result in increased transplant-related mortality, it was observed that the cytotoxic T cells transferred in the graft also provided improved anti-cancer (leukemia) activity (1). These seminal discoveries led to investigation of cellular therapies in clinical modalities, including infusion of tumor infiltrating lymphocytes (TIL) for greater disease control [reviewed in (2)].

The cytotoxic activities of immune cells, like T and NK cells, can be exploited to generate more effective anti-cancer cell therapies. In the case of T cells, the T cell receptor (TCR) is activated upon recognition of and binding to “foreign” peptides presented by the major histocompatibility complex (MHC) class I on antigen presenting cells. A cascade of signaling events then ensues, including co-receptor binding that leads to activation of the SRC tyrosine kinase LCK, which phosphorylates immunoreceptor tyrosine-based activation motifs (ITAMs) in the CD3ζ complex. ZAP-70 is recruited to the phosphorylated CD3ζ and orchestrates downstream signaling events that lead to NFAT and AP-1 activation, resulting in T cell expansion, cytokine production (e.g., IL2, IFNγ) and stimulation of cytotoxic activity (3, 4). To avoid detection and subsequent elimination by T cells, transformed cells often exhibit repressed levels of MHC expression (5, 6). In contrast, NK cells become activated depending on the balance of activating and inhibitory signals that are generated by NK cell receptors during surveillance of cells that they contact (7). As loss of MHC on a tumor cell results in decreased inhibitory signaling in the NK cell, cancer cells must use other mechanisms to inhibit the cytotoxic function of NK cells. Alternative tumor immune escape mechanisms include upregulation of HLA-E on the tumor cell surface and release of soluble NKG2D ligands, such as MICA and MICB (8–10).

**FIGURE 1:**
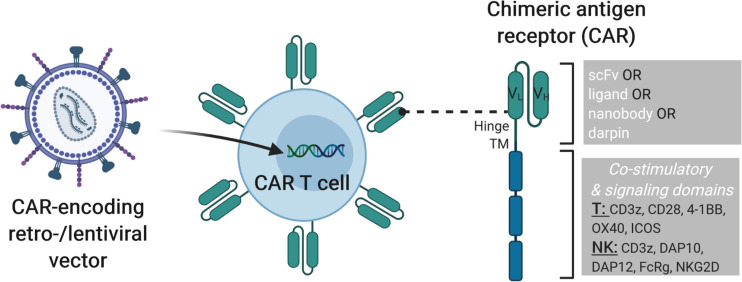
Modification of T or NK cells with CAR-encoding retro- and lentiviral vectors. On the left, a lentiviral vector is shown that transfers the genetic cargo into the T or NK cells leading to the expression of a chimeric antigen receptor (CAR) on the cell surface. On the right, the structure of a 3^*rd*^ generation CAR is depicted with single chain variable fragment (scFv, including V_*H*_ and V_*L*_ chains), hinge, transmembrane and signaling domains shown. CARs can be engineered with cell-type specific modules to enhance CAR T or CAR NK cell activity. Examples of cytoplasmic signaling domains that can be engineered into CARs for T and NK cells are shown. Combining such strategies with additional genome modification approaches described in later sections of this review will lead to improved “off-the-shelf” cell products.

The idea of combining the anti-cancer activity of immune cells, such as T and NK cells, with the concept of antibody-specificity to redirect the cytotoxic activity of these cells to target tumor cells that express a particular antigen led to the development of chimeric antigen receptor (CAR) T and NK cells (11). Specifically, CARs are synthetic receptors that contain an extracellular antibody-like region designed to target a specific antigen called the single chain variable fragment (scFv), a hinge region that can be of different lengths, the choice of which may be guided by the proximity of the recognized epitope to the target cell surface, a transmembrane domain, one or more co-stimulatory domains and a signaling domain to induce cytotoxicity upon antigen binding (Figure 1). The choice of the co-stimulatory and signaling domains have been largely based upon components of the T cell receptor (TCR), i.e., containing CD28 and/or 4-1BB costimulatory domains and a CD3ζ signaling domain. Clinically approved second generation CARs contain a CD3ζ signaling domain in combination with either a CD28 (Yescarta^®^) or 4-1BB (Kymriah^®^) co-stimulatory domain. While the CD28-CD3ζ-containing CAR T cells were shown to exhibit more rapid and stronger signaling and to favor development of effector cell phenotypes, 4-1BB-CD3ζ-containing CAR T cells had a memory cell phenotype with greater persistence (12). Direct comparison of CD28 and 4-1BB co-stimulatory domains in anti-CD19-CARs showed that 4-1BB contributes to greater CAR T cell persistence and a more favorable toxicity profile in B cell non-Hodgkin’s lymphoma (B-NHL) patients (13). The efficacy of CAR T cells, and recently CAR NK cells, has been shown for liquid tumors, most prominently in CD19^+^ lymphoid-derived cancers and several clinical studies currently explore the translation of these promising results to solid tumors (link to these studies on clinicaltrials.gov). However, there are important clinical challenges that must be addressed to further improve CAR T cell approaches. For example, one major adverse event that commonly occurs during CAR T cell therapy is cytokine release syndrome (CRS), in which greatly elevated levels of inflammatory cytokines such as interleukin (IL)-6 are observed. Severity of CRS was correlated with patient IL-6 levels and the anti-IL-6 receptor antibody tocilizumab can be used to reverse CRS symptoms without interfering with CAR T cell anti-tumor activity (14). CRS was more severe in B-NHL patients treated with CD28-CD3ζ CAR T cells as compared to 4-1BB-CD3ζ CAR T cells, possibly due to the high immune response induced by CD28 stimulation (13). As it is not always possible to identify a neoantigen that is only expressed on the tumor cell to be targeted by a CAR-modified cell, healthy cells may also be eliminated by on-target-off-tumor activity. While this may be clinically manageable in some cases, e.g., loss of healthy B cells with CARs directed against CD19, the adverse events due to on-target-off-tumor activity may be more severe with other targets, such as unwanted destruction of lung tissue after administration of anti-ERBB2(HER2)-CAR T cells designed to treat metastatic ERBB2^+^ cancer (15). The severity of on-target-off-tumor activity may be modulated by the dose of CAR T cells applied, as another study that tested HER2-CAR T cells in sarcoma patients showed this to be safe if administered up to 1 × 10^8^ CAR T/m^2^ compared to 10^10^ (or 6.25 × 10^10^ based on average female body surface area of 1.6 m^2^) CAR T cells in the former study (16). Disease relapse due to lack of CAR T cell persistence has also been reported. Loss of anti-CD19 CAR T cells was found to result from CD8^+^ immunity against the CAR T cells in some patients, which may have been due to the use of a murine scFv in the clinical CAR construct (17). To decrease potential immunogenic effects of CAR scFv sequences derived from mouse monoclonal antibodies, and, thus improve CAR T cell persistence, these should be humanized (18) (NCT02659943). To increase safety of CAR T cell therapies, vectors designed to deliver the CAR can be engineered to co-express suicide genes to allow removal of CAR T cells in case of uncontrollable severe adverse events. Examples of clinically available suicide gene strategies include the HSV-tk suicide gene (19), which makes the cells sensitive to ganciclovir-induced cytotoxicity or the inducible caspase 9 (iCasp9) gene cassette, which leads to rapid caspase-mediated apoptosis of expressing cells (e.g., CAR T cells) upon application of a synthetic inducer of dimerization, such as AP1903 or AP20187 (20, 21).

T and NK cells engineered to express CARs still eliminate target cells via the same cytotoxic mechanisms as unmodified T and NK cells, i.e., by release of perforins and granzymes, as well as death receptor interactions (22, 23). However, the cytotoxic activity is specifically amplified through binding of the scFv to the respective tumor-associated antigen. In addition, the concept of CAR T cell therapy is also applicable to other disease indications, incl. autoimmune diseases, in which CARs are introduced into regulatory T cells (Tregs), which have anti-inflammatory activities [(24) and reviewed in (25)].

Development of cell-based immunotherapeutic treatment strategies is, at least partially, directed by the characteristics of the disease to be treated and the available technology or feasibility to generate the necessary technologies. In the case of generating new CAR therapeutics to treat cancer, one of the key decisions to be made is which tumor-associated antigens to target with the scFv design. This will largely determine the specificity of tumor targeting and the extent of on-target, but also off-tumor side effects. Another important consideration is the design of the remaining domains of the CAR, for example which transmembrane, co-stimulatory and signaling domains should be incorporated. This decision may also be influenced by the cell type (e.g., T cell, NK cell, other immune cells) to be used as the “living” drug as well as the temporal window in which these cell therapies should be active. Interestingly, CAR designs based on the T cell receptor also function in NK cells (26–28). However, this does not rule out the possibility to engineer immune cell type-specific CARs for optimal use in the chosen cell type (Figure 1). For example, modification of NK cells with a chimeric receptor consisting of the NK cell activating receptor NKG2D, DNAX-activation protein 10 (DAP10) and CD3ζ led to increased cytotoxic activity against cancer cell lines and improved activity in an osteosarcoma mouse model (29, 30). CAR NK cells designed to target the prostate stem cell antigen (PSCA) on prostate cancer cells were modified with a CAR vector in which the CD28 transmembrane and costimulatory domains as well as the CD3ζ signaling domain were exchanged for DNAX-activation protein 12 (DAP12) transmembrane and intracellular signaling domains, which resulted in specific cytotoxicity against PSCA-positive tumor cell lines as compared to PSCA-negative tumor cells *in vitro* and *in vivo*. NK cells modified with a chimeric receptor that fused NKG2D to CD3ζ (NKG2D.ζ) eliminated myeloid-derived suppressor cells (MDSC) and the anti-cancer activity of these modified NK cells was not suppressed by the tumor microenvironment (TME) (31). Of note, CAR T cells that were administered following NKG2D.ζ-NK cells had improved tumor infiltration and anti-cancer activity. While most studies to date collect and modify autologous T cells to produce CAR T cell therapies, use of allogeneic CAR NK cells derived from primary NK cells (e.g., cord or peripheral blood) or from NK cell lines derived from lymphoma patients (e.g., NK-92) is increasing and other “off-the-shelf” cell sources are also being tested.

In the following sections, important concepts of how to generate “off-the-shelf” CAR cell therapies, such as the source of immune cells to be modified, strategies to overcome tumor immune escape mechanisms and genome engineering approaches that can be applied to improve CAR T and CAR NK cell function will be considered.

## T Cell Sources: Autologous, Allogeneic, Induced Pluripotent Stem Cell-Derived and Expanded Progenitor-Derived

The most common source of CAR T cells currently applied clinically is patient-derived autologous T cells, which are then genetically modified to express the CAR of choice, expanded and re-infused into the patient. Lentiviral or gammaretroviral vectors are often used in clinical trials to deliver the CAR into the T cell genome (Figure 1) (32, 33), but also non-viral integrating technologies such as Sleeping Beauty transposons were shown to efficiently generate CAR T cells (34). While use of autologous cells is enticing as this avoids challenges with immuno-incompatibilities, such as complications like GVHD, there are also disadvantages with autologous cell sources. For example, the immune cell populations may be adversely affected in heavily pre-treated patients so that the quality and number of cells for *ex vivo* modification and expansion may be suboptimal. Additionally, patients who have infections or rapidly advancing cancers might not survive the several weeks needed to produce autologous CAR T cells, as the cells have to be collected by apheresis, shipped to the facility site for genetic modification, expansion and formulation, before being shipped back to the hospital where the patient will be infused with the CAR T cells. Advantages of allogeneic CAR T cells include a lower risk of genetically modifying and re-infusing leukemic cells (35), and allogeneic cells can be prepared and stored for future use so that there is a shorter waiting period for infusion into the patient. Thus, “off-the-shelf” allogeneic cell sources could provide greater flexibility for treatment protocols, potentially lower overall costs if multiple patients can be treated from a single CAR T cell product and could be expected to allow broader access to these clinical procedures (36).

Therefore, methods for efficient and reliable production of “off-the-shelf” T cells remain highly sought goals in the field of cellular immunotherapy. Important conditions that these cells must meet include avoidance of rejection due to recognition by host T cells via HLA class I molecules or host NK cells by HLA class II receptors. “Off-the-shelf” immune cells should also lack alloreactivity to limit unwanted toxicities due to recognition and destruction of healthy host tissues. Several strategies to avoid host cell recognition have been explored, such as knock-down or knock-out of MHC molecules to block recognition by host T cells. However, this can result in elimination of the modified cells due to NK cell activity against cells lacking MHC expression (37–39). Expression of ligands that inhibit NK cell cytotoxicity, like HLA E or HLA G, can ameliorate elimination of the engineered cells, but since receptors for these ligands are not expressed on all NK cells, veritable “off-the-shelf” cell therapeutics will likely require genetic engineering strategies that address multiple layers of immune cell recognition patterns and cytotoxic mechanisms (40–42). Importantly, these modifications would ideally not negatively impact immune cell expansion that is necessary for clinical application or cell persistence and function, which are important for immune cell anti-cancer efficacy. Additionally, potential risks of genetically modified immune cells must be evaluated, although such risks are generally low in differentiated somatic cells like T cells.

## NK Cell Sources

An alternative approach is to exploit the natural cytotoxic activity of NK cells to generate allogeneic “off-the-shelf” CAR NK cells to target cancer cells. One advantage of NK cells is that they were shown to not induce GVHD even in mismatched settings (43). However, an earlier study observed acute GVHD in five of nine patients who received donor-derived allogeneic NK-donor lymphocyte infusions (NK-DLI) after HLA-matched transplantation of T cell-depleted (for delivery of ≤ 2 × 10^4^ T cells/kg) peripheral blood stem cells from matched sibling donors (1 of 5) or matched unrelated donors (4 of 4) (44). The authors propose that allogeneic NK-DLI may have contributed to the observed GVHD by aggravating an existing subclinical T cell-mediated GVHD. This is supported by the assessment of donor chimerism based upon CD3, which showed significantly higher donor chimerism in GVHD patients, and that allogeneic NK-DLI was accomplished shortly prior to detection of the high donor chimerism in three of the five patients who developed GVHD. The relative risk of GVHD following NK cell application will become clearer as more data accumulates with CAR NK cells, which are increasingly incorporated into clinical trials (Table 1).

**TABLE 1:** Selected clinical trials testing potential “off-the-shelf” CAR cell therapies.

Cells	CAR	Diseases/Patients	1°/2° outcomes	References	
**No genome modification**					
Allogeneic T cells	CD19-CAR	Relapsed or refractory CD19^+^ B cell malignancies	1°: DLT, CR 2°: ORR, DOR, safety, tolerability, TRM	NCT04384393	ThisCART19
Allogeneic T cells	CD19-CAR	Elderly relapsed or refractory B-ALL	1°: occurrence of adverse events 2°: overall response rate, DFS, OS	NCT02799550	
Allogeneic T cells	alloCART-19	Pediatric relapsed or refractory ALL	1°: DLT 2°: AE, ORR, BOR	NCT04173988	
Allogeneic T cells	NKG2D-based CAR-T plus inhibitory peptide T cell receptor (TCR) inhibiting molecule (TIM) to reduce signaling of the TCR complex through a non-gene edited approach	Metastatic colorectal cancer (mCRC)	1°: DLT, ORR 2°: AE, safety, ORR, BOR, kinetics, clinical activity, PFS, EFS, OS	NCT03692429	alloSHRINK trial
**Genome modified**					
Allogeneic T cells (TCRα/β disruption)	Anti-CS1 CAR (UCARTCS1A)	Relapsed or refractory MM	1°: safety	NCT04142619	MELANI-01
Allogeneic T cells (TCRα/β disruption)	Anti-CD123 (UCART123)	Relapsed or refractory AML	1°: safety, tolerability	NCT03190278	AMELI-01
Allogeneic T cells (TCRα/β disruption)	CD19-UCART	Relapsed or refractory B cell malignancies	1°: DLT 2°: ORR, CART persistence	NCT03229876	
Allogeneic T cells (TCRα/β disruption)	BCMA-UCART	Relapsed or refractory MM	1°: ORR 2°: safety, tolerability, CART persistence	NCT03752541	
Allogeneic T cells (TCRα/β disruption)	CD22-CAR (UCART22)	Relapsed or refractory CD22^+^ B-cell B-ALL	1°: safety, tolerability	NCT04150497	BALLI-01
Allogeneic T cells (TCRα/β disruption)	CD19-UCART	Relapsed or refractory B-ALL	1°: DLT 2°: safety, tolerability, objective remission rate and duration, PFS, OS	NCT02746952	CALM
Allogeneic T cells (TCRα/β and B2M disruption)	UCART019	Relapsed or refractory CD19^+^ leukemia and lymphoma	1°: safety, feasibility, persistence 2°: tumor response, test for humoral immunity against murine CD19 scFv	NCT03166878	
Allogeneic T cells (TCRα/β and B2M disruption)	CTX110 (CD19-CAR)	Relapsed or refractory B cell malignancies	1°: DLT, ORR 2°: DOR, PFS, OS	NCT04035434	
Allogeneic T cells (TCRα/β and B2M disruption)	CTX120 (BCMA-CAR)	Relapsed or refractory MM	1°: AE, DLT, ORR 2°: PFS, OS	NCT04244656	
Allogeneic T cells (TCRα/β and B2M disruption)	CTX130 (CD70-CAR)	Relapsed or refractory renal cell carcinoma	1°: AE, DLT, ORR 2°: PFS, OS	NCT04438083	
Allogeneic T cells (TCRα/β and B2M disruption)	Universal Dual CD19 + CD20-CAR or CD19 + CD22-CAR	Relapsed or refractory B-cell malignancies	1°: safety, feasibility, persistence 2°: anti-tumor response, test for humoral immunity against murine CD19 scFv	NCT03398967	
Donor T cells (CMV- or EBV-specific T cells derived from donor CD62L + TCM cells)	CD19-CAR	B cell malignancies after allogeneic transplant	1°: safety, feasibility 2°: persistence, trafficking to bone marrow, function, CMV/EBV reactivation, elimination of CD19^+^ tumor cells	NCT01475058	
Allogeneic EBV specific cytotoxic T-lymphocytes (EBV-CTLs)	CD19-CAR	B cell malignancies after allogeneic transplant or high risk for relapse	1°: safety, persistence 2°: assess effects on leukemia progression, CAR-T cell survival and	NCT01430390	
			proliferation, long-term status of treated patients		
Allogeneic EBV specific T cells	Anti-CD30 CAR	Relapsed or refractory CD30^+^ lymphoma	1°: DLT 2°: ORR, DOR, SD, PFS	NCT04288726	
**Other “off-the-shelf” CAR cells**					
NK-92 cell line	CD33-CAR (CD28-CD137 (4-1BB)-CD3ζ)	Relapsed or refractory CD33^+^ AML	1°: safety, feasibility 2°: anti-leukemia response, *in vitro* anti-AML cytotoxicity, test for development of humoral immunity against the murine anti-CD33 scFv	NCT02944162	
NK-92 cell line	CD7-CAR (CD28-4-1BB-CD3ζ)	CD7 + leukemia and lymphoma	1°: AE, toxicity profile 2°: clinical response, persistence	NCT02742727	
NK-92 cell line	CD19-CAR	CD19^+^ leukemia and lymphoma	1°: AE 2°: ORR	NCT02892695	
Allogeneic NKT cells	CD19-CAR + IL-15	Relapsed or refractory B cell malignancies	1°: DLT 2°: persistence of modified cells, overall response	NCT03774654	ANCHOR
Haploidentical/Allogeneic Gamma Delta (γδ) T cells	NKG2DL-targeting CAR	Relapsed or refractory solid tumors	1°: DLT 2°: AE, efficacy, PFS, DOR	NCT04107142	
Allogeneic Gamma Delta (γδ) T cells	CD19-CAR	High risk, relapsed CD19^+^ B cell malignancies	1°: safety (adverse events) 2°: CAR γδ persistence, antitumor activity, MTD	NCT02656147	

*AE, adverse events; ALL, acute lymphoblastic leukemia; AML, acute myeloid leukemia; B-ALL, B-cell acute lymphoblastic leukemia; BOR, best overall response; CR, complete remission; DFS, disease-free survival; DLT, dose limiting toxicities; DOR, duration of response; EBV, Epstein Barr virus; MM, multiple myeloma; ORR, objective response rate; PFS, progression-free survival; OS, overall survival; scFv, single chain variable fragment; SD, stable disease; TRM, treatment related mortality.*

Different NK sources have been used to generate pre-clinically and clinically tested CAR NK cells, including cell lines such as NK-92 cells (45), cord blood-derived NK cells (43, 46) and peripheral blood-derived NK cells (28). Of note, a recent landmark phase 1 and 2 study showed the feasibility of cord-blood-derived CAR NK cells to treat relapsed or refractory CD19^+^ B-cell cancers (43). Eight of eleven (73%) patients responded rapidly (within 30 days after CAR NK cell infusion), including seven complete remissions. Of particular interest, the only major adverse events were related to the lymphodepletion strategy (i.e., neutropenia, lymphopenia) and no cytokine release syndrome, neurologic events or GVHD were observed, even with 2-5 HLA allelic mismatches (43). CAR NK cells persisted for at least 12 months after infusion, which may have been at least partially due to inclusion of an IL-15 expression cassette in the CAR construct, a cytokine known to enhance NK cell survival and proliferation (43). The same group previously showed that one cord-blood unit could be used to produce over 100 CAR NK doses, further highlighting allogeneic CAR NK cells as potential “off-the-shelf” drugs (47). While regulatory guidelines may vary depending on the country in which the study is performed, cell therapeutics should be viable (e.g., ≥70%) and demonstrated to be negative for endotoxin, mycoplasma or bacterial contaminations. For CAR NK cells, the cell product should contain mostly CD56^+^ cells (≥90%), and be free of CD3^+^ cells (e.g., ≤0.2%) and CD14^+^ cells (e.g., ≤5%). In the case that CAR NK cells are expanded via co-culture with irradiated feeder cells, for example, membrane bound IL-15 and 4-1BB ligand expressing K562 cells or membrane bound IL-21 expressing OCI-AML3 cells, the final CAR NK cell product should be demonstrated to be free from contamination of co-cultured cells (e.g., ≤1%) (48, 49). Contamination of primary NK cell therapeutics with feeder cells may be mitigated by alternative expansion methods, such as use of coated beads or cytokine combinations to expand NK cells. Primary NK cells can be activated and expanded with cytokines such as IL-2, IL-12, IL-15, IL18, and IL-21 (50–53). Similarly to expansion of primary T cells with CD3/CD28 beads, primary NK cells can also be expanded with CD335 (NKp46)/CD2 beads.

## iPSC and other Cell Sources

Additional cell sources to produce “off-the-shelf” CAR cells include stem cell and progenitor cell populations such as induced pluripotent stem cells (iPSC) and precursor T cells. iPSC possess a nearly unlimited proliferative potential and can be differentiated into various cell types, including T and NK cells. Thus, iPSC offer a renewable source of potentially standardized cells for immunotherapies and can be easily genetically modified to generate immune cells with improved characteristics (54). The feasibility of producing CAR T cells from iPSC was demonstrated by transduction of peripheral blood lymphocyte-derived iPSC with a lentiviral vector encoding for a second-generation anti-CD19-CAR (55). After hematopoietic specification and expansion, the authors used a T-lymphoid commitment co-culture protocol to generate anti-CD19-CAR-T-iPSC-T. The authors directly compared the iPSC-derived CAR T cells with TCR-αβ and TCR-γδ peripheral blood lymphocytes from the same donor and transduced with the same CAR and demonstrated that the iPSC-derived CAR T cells showed a similar anti-cancer activity as the CAR TCR-γδ cells in an immunodeficient mouse xenograft tumor model using the CD19^+^ Raji human Burkitt lymphoma cell line (55).

Adaptation of CAR designs to exploit the signal transduction pathways naturally used for cell activity may lead to improved CAR NK or other CAR-cell type activities. For example, an “NK-CAR” engineered to contain the NKG2D transmembrane domain, the 2B4 co-stimulatory domain and the CD3ζ signaling domain was used to modify iPSC cells, which were subsequently differentiated into NK-CAR-iPSC-NK cells (iPSC-derived NK cells equipped with an NK-CAR). The NK-CAR-iPSC-NK cells demonstrated superior anti-tumor activity when directly compared to T-CAR-iPSC-NK cells in an ovarian cancer xenograft model and had similar activity as observed for CAR T cells that expressed a typical CAR designed for T cells (CD28-CD3ζ) (56). Advantages of iPSC-derived CAR T/CAR NK cells include their enormous proliferative and expansion capacities as well as the relative ease of genomic modification, which provides the possibility to create cell banks with different CAR constructs as standardized “off-the-shelf” immunotherapies.

A recently described inducible transcription factor-mediated forward programming approach to efficiently produce large numbers of hemato-endothelial progenitor cells and hematopoietic progenitor cells may also become useful for generating “off-the-shelf” cell therapies, such as CAR NK cells (57). While this strategy led to sustained production of myeloid lineages, differentiation into the lymphoid lineages was less robust. However, RNAseq interrogation of gene expression patterns revealed several transcription factor targets whose expression could potentially be modulated to overcome this. Similarly, methods to produce conditionally immortalized murine lymphoid progenitors might be exploited to efficiently generate CAR T cells, although this remains to be tested and applied to human lymphoid progenitors (58, 59). Expression of an anti-CD19-CAR in lymphoid progenitors was shown to suppress T cell development with the generation of cells with NK cell-like characteristics that had strong cytotoxic activity against CD19^+^ leukemia cells across MHC barriers and without causing GVHD. Importantly, this shift in differentiation was dependent upon ongoing signaling activity of the respective CAR early during hematopoietic development (60). Most recently, CAR-macrophages (CAR-Ms) were shown to phagocytose tumor cells in an antigen-specific manner, decrease tumor burden in two solid tumor xenograft mouse models and to promote anti-cancer T cell activity by inducing a pro-inflammatory tumor microenvironment (61).

Thus, much progress has been made in identification of alternative “off-the-shelf” therapeutic CAR cells. As any manipulation of the genome, such as insertion of therapeutic CAR vectors (Figure 1), carries an inherent risk, these must be carefully evaluated. While possible genotoxic risks such as transformation of a healthy cell to a cancer cell are low in terminally differentiated somatic cells like T and NK cells, such modifications in stem cells (e.g., HSC, iPSC) or progenitor populations that can be differentiated into T or NK cells may carry higher risks, which should be assessed and mitigated as necessary. There are also differences in regulatory requirements for clinical use of primary lymphocytes and cell lines. For example, cell lines must be irradiated to minimize the risk of secondary lymphoma (e.g., due to uncontrolled proliferation of immortalized cell lines), and it has to be documented that culture conditions did not include animal based supplements (e.g., fetal bovine serum) or antibiotics (e.g., penicillin, streptomycin).

## Genome Modification Techniques and Application to Car T Cells

In addition to gene transfer technologies to improve CAR T cell function, genomic modification strategies have been used to advance “off-the-shelf” cell therapeutics. Zinc-finger nucleases (ZFN), transcription activator-like nucleases (TALENs) and clustered regularly interspaced short palindromic repeats (CRISPR)-Cas9 (CRISPR-associated protein 9) systems are currently the most commonly employed genome editing technologies. ZFN and TALEN technologies target specific genomic loci via protein-DNA interactions, and require protein engineering expertise that is not available in every laboratory. In contrast, the CRISPR-Cas9 system uses RNA-guided DNA recognition to define the genomic modification locus, which makes tailored design of CRISPR-Cas9 to target specific genes relatively easy and has led to wide-spread use of this technique in the scientific community. Genome editing occurs after double strand break (DSB) induction through two main DNA repair mechanisms, non-homologous end joining (NHEJ) and homology directed repair (HDR), with NHEJ active throughout all phases of the cell cycle and the less efficient HDR mainly confined to the S phase. The efficiency of targeted insertion by HDR can be improved by TP53 inactivation to block the TP53 damage response and interruption of the cell-cycle induced by DNA double-stranded breaks caused by CRISPR-Cas9 (62–64). Interestingly, NHEJ was further improved by a new method called CRISPR-HOT, which stands for CRISPR–Cas9-mediated homology-independent organoid transgenesis (65). This method enables the efficient generation of knock-in human organoids in different tissues and achieves precise integration of exogenous DNA sequences into desired loci, without the necessity to inactivate TP53.

### ZFN “Off-the-Shelf” Cell Products

In order to produce “off-the-shelf” allogeneic CAR T cell products, it is necessary to disrupt adverse reactions such as GVHD due to endogenous TCR activation that occurs with HLA-mismatched donors and recipients. Possible gene editing strategies to circumvent this include elimination of endogenous TCR expression to generate universal donor T cells (66) and targeted insertion of CAR into the TCR alpha constant (TRAC) locus (67). Efficient genome editing of CD8 and CD4 T cells by HDR was accomplished using Adeno-associated virus (AAV) serotype 6 vectors (AAV6) to deliver the homologous donor template and electroporation of ZFN mRNA (68).

Electrotransfer was also used to deliver designer ZFN to delete TCR α or β chains in CD19-CAR T cells, and the TCR^–^CAR^+^ population maintained CD19 specificity without responding to TCR stimulation (69).

### TALEN “Off-the-Shelf”Cell Products

Universal CAR19T (UCART19) cells were generated using TALENs to target the constant region of the *TCR*α *chain* (*TRAC*) and the *CD52* gene to make UCART19 cells resistant to Alemtuzumab (Campath^®^), which is an antibody used to eliminate CD52^+^ lymphocytes in B-cell chronic lymphocytic leukemia (66). UCART cells led to rapid molecular remissions (28 days) in two infants with refractory high-risk B-ALL. One patient had grade 2 skin GVHD and the second patient had a possible mild skin GVHD that was quickly resolved with topical steroids (66). TALENs were also used to disrupt the TCRαβ locus to generate universal allogeneic CAR T cells directed against the tumor-associated antigen CS1 (UCARTCS1A), which are currently tested in relapsed and refractory multiple myeloma patients (NCT04142619) (Table 1). A similar approach was used to generate universally applicable anti-CD22 CAR T cells (UCART22) to treat patients with relapsed and refractory CD22^+^ B-cell B-ALL (NCT04150497).

### CRISPR-Cas9 “Off-the-Shelf”Cell Products and Developing Technologies

CRISPR-Cas9 RNPs and AAV6 were used to specifically deliver an engineered 2.3-kb-long TCR construct TCR25D6, which recognizes a peptide derived from myeloperoxidase as a tumor-associated antigen in myeloid neoplasia patients when presented on HLA-B7, into the *TRAC* locus (70). CRISPR-Cas9-mediated knockout of TCRα/β and B2M in combination with lentiviral delivery of an anti-CD19 CAR into allogeneic T cells resulted in universal CAR T cells (UCART019) that are clinically tested in relapsed or refractory CD19^+^ leukemia and lymphoma patients (NCT03166878). CRISPR-Cas9-mediated TCRα/β and B2M knockout to generate “off-the-shelf” allogeneic CAR T cells is also evaluated in other clinical trials for CD19^+^ leukemia and lymphoma patients (NCT04035434), multiple myeloma patients (NCT04244656) and renal cell carcinoma patients (NCT04438083) (Table 1).

Multiplex CRISPR-Cas9 allows simultaneous editing of several genomic loci. The feasibility and safety of using multiplex CRISPR-Cas9 to engineer autologous T cells with enhanced anticancer activity was recently demonstrated in a phase I trial (NCT03399448) (71). CRISPR guide RNA was electroporated into T cells to delete endogenous TCRα and TCRβ chains as well as the *PDCD1* gene that encodes the programmed cell death protein 1 (PD-1). Endogenous TCR disruption was done to allow enhanced expression of the cancer-specific TCR NY-ESO-1, which was introduced by lentiviral transduction. In addition, PD-1 knockout was accomplished to improve activity of the engineered T cells by avoiding checkpoint inhibition through tumor-associated cells. This may be an important strategy as disruption of PD-1 on T cells may help avoid immune-related side effects observed upon systemic administration of anti-PD-1 monoclonal antibodies, while still improving CAR T cell anti-tumor activity.

### Advances to Increase CRISPR Technology Specificity and Safety

**FIGURE 2:**
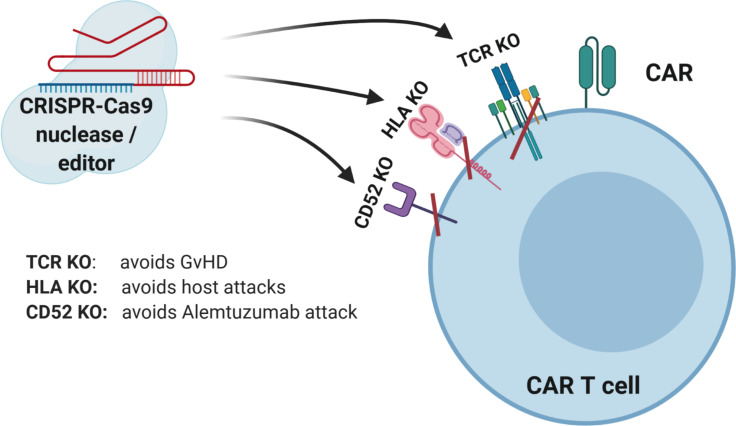
CRISPR-Cas9-mediated gene editing of CAR T cells. The TCR can be knocked out to lessen the likelihood of graft versus host disease (GVHD). The HLAs can be knocked out to increase persistence of gene-modified cells. Knockout of receptors that can be targeted by other medications, such as antibodies, can be accomplished to allow selective survival of gene-modified cells, e.g., CD52.

While RNA-guided (sgRNA) programmable nucleases based on CRISPR-Cas9 are very versatile and useful tools and mostly generate accurate and precise DNA DSBs, potentially also off-target effects can occur. Furthermore, chromosomal translocations are rare unwanted side effects, especially in case of multiplexing (72). To decrease the risk of these unwanted events, further engineering, e.g., CRISPR-Cas9 systems with less off-target effects, and newer gene editing approaches are being developed as discussed below. Such advances will lead to more efficient and safer generation of genome modified “off-the-shelf” CAR T and CAR NK cell products (Figure 2).

CRISPR-Cas9 has two nuclease domains and introduction of inactivating mutations into one of these domains results in so-called nickases, which cleave only one strand of the targeted DNA (73). As a further improvement, “dead” Cas9 variants with inactivating mutations in both nuclease domains were created that could be fused to DNA modifying enzymes, such as Apobec-like nucleobase deaminase enzymes. These “base editors” lead to defined base alterations without the need to cut the DNA and thus further reduce the likelihood of side effects (74). More recently, a catalytically impaired Cas9 was fused to an engineered (Murine Leukemia Virus-derived) reverse transcriptase to enable “prime editing” as a new technology to write new genetic information into a specified DNA site (75). Accordingly, prime editing further expands the capabilities of gene editing to create new options for immunotherapeutics.

Additional approaches have been developed with the intent to minimize possibly deleterious activity of genome editing described above. For example, as long-term CRISPR-Cas9 expression is not required for efficient genome modification, transient RNA-protein (RNP) complexes can be delivered into the target cell population in place of viral vectors or DNA constructs. Alternatively, non-integrating lentiviral vectors can be engineered for transient delivery of CRISPR-Cas9 editing coupled with the possibility to target specific cell populations (76). High-fidelity CRISPR-Cas9 nuclease variants designed to have fewer interactions with non-specific DNA sequences, but maintaining on-target DNA activity were also developed (77). As mentioned above, novel Cas9 fusion proteins were engineered to create base editors, i.e., cytosine base editors (CBE) and adenosine base editors (ABE), capable of editing single bases (78, 79). CBE were generated by fusing a cytidine deaminase to a catalytically impaired Cas9 protein (i.e., that is unable to induce double-strand DNA breaks) and uracil glycosylase inhibitor. Since Cas9-independent off-target DNA editing was observed with CBEs largely due to cytidine deaminase activity, additional Cas9 fusion variants were generated and shown to have up to 100-fold less Cas9-independent off-target DNA editing, but retained 50–90% of on-target DNA editing (80). Approaches like these will make it easier to safely modify allogeneic T cells into universal CAR T cells via disruption of TCRα/β and B2M without the need to introduce double strand DNA breaks.

## Mechanisms to Improve Immune Cell Anti-Cancer Activity

In addition to enhancing immune cell recognition of tumor cells via CAR expression, additional modifications of CAR cells may be necessary to effectively overcome tumor cell resistance mechanisms. One mechanism tumor cells use to evade immune cell-mediated cytotoxicity is exploitation of immune checkpoint signaling, which is used to inhibit immunologic damage of “self” cells in the healthy state. Immune checkpoints are critical components of autoimmune tolerance to avoid autoimmune diseases like rheumatoid arthritis (81, 82), type I diabetes (83) and multiple sclerosis (84). Checkpoint receptors on immune cells recognize ligands expressed on cells being surveilled and activation of these immune checkpoint receptors by the ligands leads to inactivation of the immune cells. This mechanism is exploited by tumor cells, which may overexpress these ligands or induce other cells [e.g., tumor-associated macrophages (TAMs), myeloid-derived suppressor cells (MDSCs), regulatory T cells (Tregs)] within the tumor microenvironment (TME) to express checkpoint ligands to create an immunosuppressive shield throughout the TME and thus help tumor cells evade immunosurveillance (85–87). Secretion of immunosuppressive factors like transforming growth factor-β (TGF-β) by cells in the TME can directly inhibit CAR T cell cytotoxic activity and even direct differentiation of effector T cells to regulatory T cells (88–90).

Immune checkpoint molecules include cytotoxic T-lymphocyte-associated antigen 4 (CTLA4), PD-1 (PDCD1, CD279), lymphocyte activation gene 3 (LAG-3), and T cell membrane protein 3 (TIM3, HAVCR2) (91–94). Interaction of immune checkpoints with their cognate ligands results in suppression of immune cell function. Thus, tumor cells may express CD80/86 to suppress T cell activity via binding to CTLA4, or express PD-1 ligands PD-L1 (CD274) or PD-L2 (PDCD1LG2, PD-2 ligand). Accordingly, binding of LAG-3 to MHC class II or fibrinogen-like protein 1 (FGL1), or ligation of TIM3 to galectin 9, carcinoembryonic antigen cell adhesion molecule 1 (CEACAM1), high-mobility group box protein 1 (HMGB1) or the non-protein ligand phosphatidylserine was shown to negatively regulate immune cell cytotoxic activity (95–97).

Several antibodies were developed to inhibit the activity of immune checkpoint molecules and clinical anti-cancer activity was demonstrated for some of these checkpoint inhibitors. Currently, most studies have investigated checkpoint inhibition of CTLA4 and PD-1 activities (98, 99). However, strategies to inhibit LAG-3 may be even more promising as antibodies that target LAG-3 were shown to enhance cytotoxic T cell activation and may inhibit Treg-induced immunosuppressive activity as elevated levels of a LAG-3^+^ subpopulation of Tregs was found at tumor sites and in peripheral blood mononuclear cells of patients with melanoma or colorectal cancer (100). Combination of the anti-LAG-3 antibody IMP321 with paclitaxel led to improved immune responses and greater antitumor activity in metastatic breast cancer patients (101). Currently, more than 240 clinical studies are evaluating the efficacy of checkpoint inhibitors in several different treatment modalities in cancer patients (link to respective studies on clinicaltrials.gov). As CAR-modified immune cells can become functionally inactivated or depleted due to tumor escape mechanisms such as immune checkpoints, checkpoint inhibition can help promote CAR T and perhaps CAR NK cell persistence and anti-tumor activity. The efficacy of CAR T cells directed against mesothelin with concomitant CRISPR-Cas9-mediated knockout of TCRαβ and PD-1 is currently tested in a clinical trial (NCT03545815) of patients with mesothelin positive solid tumors. Such studies will help elucidate the feasibility of combining checkpoint inhibition in an “off-the-shelf” CAR T cell setting.

**FIGURE 3:**
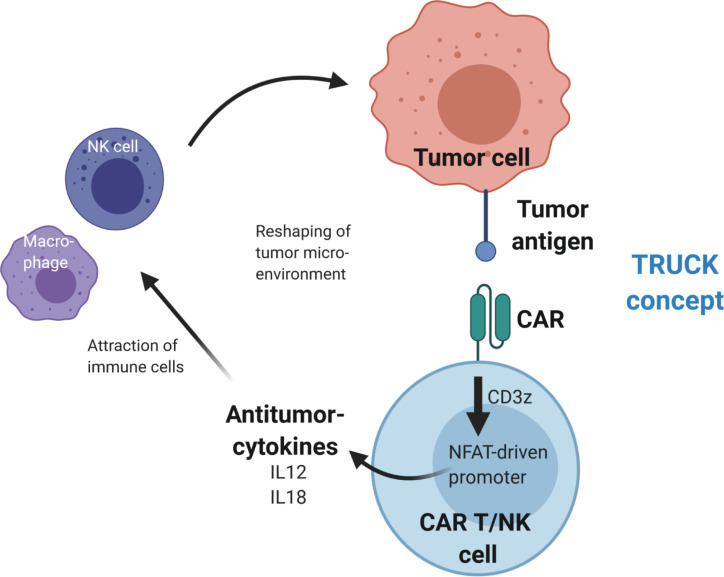
Reshaping of the tumor microenvironment using T cells redirected for universal cytokine killing (TRUCK). Upon antigen binding, the CAR activates CD3z(eta) signaling, which leads to activation of an NFAT-driven promoter that controls expression of antitumor-cytokine cassette, e.g., IL12 or IL18. The cytokines are then secreted from the CAR T or CAR NK cells into the tumor microenvironment, where they recruit additional immune cells to enhance the antitumor activity.

In addition to application of immune checkpoint inhibitors, anti-tumor activity was demonstrated by CAR-mediated cytokine secretion at the tumor site using T cells redirected for universal cell killing (TRUCKs) (102–105). This strategy involves modification of T cells with a constitutively expressed CAR and a cytokine expression cassette that is controlled by an inducible promoter. The TRUCK concept uses the NFAT signaling pathway to produce pro-inflammatory cytokines upon activation of the CAR CD3ζ signaling domain after tumor antigen recognition. This results in modification of the TME via cytokine secretion and recruitment of additional anti-tumor immune cells to increase anti-cancer activity (see Figure 3). In the original design, TRUCKs were generated by retroviral vector-mediated transfer of two separate vectors – one for the CAR and the second for the inducible cytokine expression cassette. Recent work showed the feasibility to deliver the necessary genetic cargo on a single lentiviral vector (106), thus advancing the potential use of this technology for “off-the-shelf” immunotherapy.

## Direct *In Vivo* Application of CAR-Based Principles Using Targeted Viral Vector Nano-Particles

As the whole genesis of CAR-harboring cellular products is highly demanding, future-oriented approaches for “off-the-shelf” applications are also considering the direct application of GMP-grade viral vector preparations to deliver the CAR-based principles directly into the target immune cells of choice, e.g., T and NK cells. This method entirely circumvents immunological rejection barriers, avoids time consuming *ex vivo* manipulation and cultivation of cells, and would directly reach the target effector cells of the individual. Of note, receptor-targeted vector particles can be as selective for their targeted cell type as antibodies for their antigen when applied systemically or locally in preclinical studies. In this regard, receptor targeting using viral vector nano-particles opens up the possibility for novel concepts in immunotherapy and cell type-specific delivery of CARs in *in vivo* settings (107). Similar delivery principles were shown for non-viral delivery of mRNA in lipid nanoparticles (108).

As a prerequisite for this approach, the natural tropism of the used viral vectors, e.g., gammaretro-/lentiviral and AAV vectors, needs to be blinded, so that the viral vectors no longer bind to their natural target receptors. In a second step, a defined and specified target selectivity has to be added by incorporation of a new selective target cell-binding principle, e.g., scFv antibody, peptide or DARPin (designed ankyrin repeat protein) (107, 109, 110).

### Retroviral Vectors for “Off-the-Shelf” CAR Delivery

In the case of enveloped gammaretro- and lentiviral vectors, the envelope for pseudotyping is substituted for a “targeted” Measles (111) or Nipah virus-derived envelope (112) with a newly assigned target specificity. Proof-of-concept for targeted delivery was demonstrated for a variety of target cells, including various hematopoietic and endothelial cells. Of particular importance for CAR technology, surface-engineered lentiviral vectors were successfully applied to mediate selective gene transfer into various subtypes of lymphocytes, including T cells (110, 113) that, impressively, led to the *in vivo* generation of human CD19-CAR T cells with B-cell depletion and signs of cytokine release syndrome in a humanized mouse model (114).

### AAV Vectors for “Off-the-Shelf” CAR Delivery

In the case of AAV vectors, which are derived from non-enveloped viruses, the capsid is the target of engineering. Prominent capsid structures are protrusions, which host the natural receptor binding motifs and pores used for loading of vector DNA. Genetic targeting approaches are the currently preferred strategy to modify vector tropism, and were used to insert receptor binding peptides [reviewed in (115)], immunoglobulin binding domains (116) or nanobodies (117) at the tip of the protrusions. Alternatively, the N-terminus of the non-essential capsid protein VP2 can be used as an insertion site. This is especially useful (I) to incorporate large peptides, (II) to target moieties that depend upon their 3D structure for function or (III) to incorporate entire proteins (118–122). The respective fusion proteins become exposed on the capsid surface through the pore structures. Tropism can either be expanded or re-directed, depending on the specificity of the targeting moiety that is inserted, and whether or not the natural tropism has been ablated, for example by site-directed mutagenesis. The feasibility that off-target free, on-target delivery following intravenous administration of viral vector particles is possible was demonstrated by incorporating DARPins with antibody-like specificity via fusion to VP2 into AAV2 capsids blinded for binding to their primary receptor heparan sulfate proteoglycan (109). These AAV particles can efficiently discriminate between target and non-target cells *ex vivo* in mixed cell cultures as well as *in vivo*, e.g., as demonstrated by delivery of a suicide gene precisely into tumor tissue and specific targeting of CD4^+^ lymphocytes *in vivo* (109, 122).

These systems will further enrich the portfolio of “off-the-shelf” applications for cancer immunotherapy.

## Discussion/Outlook

Several factors impact the potency and successful translation of adoptive cell therapies like CAR T and CAR NK cells to treat cancer. As discussed above, selection of the cell source is a critical decision. The majority of CAR-based therapies use autologous T cells, which have been successfully administered in several clinical studies, with broader success in hematologic malignancies (especially of the lymphoid compartment) than in solid tumors thus far. Autologous CAR T cells have advantages such as no risk of GVHD and lower risk of rejection than allogeneic CAR T cells. However, autologous CAR T cells may have some immunologic defects and the patient must wait several weeks before the autologous CAR T cells are ready for application. As we seek to extend the clinical usefulness of CAR cell strategies, one obvious path forward is to commit more resources toward development of “off-the-shelf” CAR cell therapies, such as genetically modified “universal” allogeneic CAR T cells, NK cells, iPSC and progenitor-derived cells. Universal allogeneic CAR cell therapies are derived from healthy donors, so the immune cells should function properly, and the TCR and MHC are disrupted to avoid induction of GVHD or elimination by the host T cells. While such genome editing strategies to generate “off-the-shelf” CAR T cells are already in clinical practice (66, 71), these may have higher regulatory burden to demonstrate lack of off-target hits and translocations. Another advantage of allogeneic CAR cells is that they can be prepared in advance and stored until needed, thus reducing the time a patient must wait for treatment. As CRISPR-Cas9 genome modification procedures continue to become more efficient and precise, potential risks of genome modified cell therapies will decrease. For example, advances such as base editing make it possible to specifically edit the genome without the necessity to induce double strand DNA breaks, thus potentially increasing the safety of genome editing by reducing the risk of unwanted complications like chromosomal translocations in cell therapies. However, detection of off-target hits is even more difficult and will become even more challenging with advances like epigenome editing technologies.

Combinations of cell therapies may also be useful in this context, especially considering the complex interactions between different cell types during immune responses. For example, CAR T cell activity against colorectal cancer cells was recently shown to be improved by co-application of mesenchymal stem cells (MSC) genetically modified to release IL7 and IL12 (123). The authors exploited the natural capacity of MSC to home to tumor sites and thus support CAR T cells. Cross-talk between CAR T and MSC led to a greater persistence of CAR T, less activation-induced cell death and better anti-tumor activity as shown in *in vitro* and *in vivo* models. Therefore, generation of master cell banks of different types of universal allogeneic cells available as “off-the-shelf” living drugs may help increase the efficacy of immune cell therapies.

Therapeutic efficacy can be limited by loss of CAR T/CAR NK cell persistence due to rejection. As discussed above, humoral responses raised against murine-derived scFv may lead to loss of CAR-modified cells. In such cases, strategies to humanize the scFv can result in greater CAR cell persistence. Genetic ablation of MHC may also help to increase CAR cell persistence by evading the host T cell responses, but may also lead to increased detection by host NK cells.

Development of strategies to overcome tumor-induced immune suppression has been widely studied and use of immune checkpoint inhibitors or genetically engineering CAR T and CAR NK cells to be less responsive to checkpoint signaling are two main approaches to address this challenge. For example, CRISPR-Cas9-mediated elimination of the checkpoint receptor PD-1 from CAR T cells led to improved activity against the solid tumor glioblastoma in preclinical models (124). The possibility to simultaneously and efficiently modify multiple genes with CRISPR-Cas9 seems to be an advantage over ZFN and TALEN genome editing technologies.

Emergence of or selection for tumor cells that do not express the target antigen, a concept called “antigen loss,” can also negatively impact CAR T and CAR NK cell antitumor activity. For example, relapsed/refractory B-cell acute lymphoblastic leukemia (B-ALL) patients who were previously administered blinatumomab, a bispecific antibody that targets CD3 on T cells and CD19 on B cells, were less likely to achieve minimal residual disease deep remission and were more likely to experience relapse due to antigen loss after treatment with anti-CD19 CAR T cells (125). Use of dual CAR concepts to target two tumor-associated antigens can lead to improved tumor control. However, the increased risk of on-target-off-tumor activity has to be taken into account.

In addition to antigen loss to avoid CAR T cell activity, decreased death receptor activity (FADD, BID, CASP8 and TNFRSF10B) was shown to be a mechanism of resistance to anti-CD19 CAR T cells (126). Importantly, pre-treatment leukemia-infiltrated bone marrow samples from patients who were treated with anti-CD19 CAR T cell therapy showed that lower death receptor gene expression associated with worse overall survival (126). Methods to restore or elevate death receptor expression and signal transduction activity in tumor cell target populations could lead to improved tumor control. Identification of target molecules that are more specifically expressed on tumor cells and absent on healthy tissues will also increase the therapeutic efficacy of CAR T and CAR NK cell therapies. The recently described “Sequentially Tumor-selected Antibody and antigen Retrieval (STAR)” method led to isolation of nanobodies that preferentially bound acute myeloid leukemia (AML) cells, and identified CD13 as an AML-specific target (127). Generation of bi-specific CAR T cells that targeted CD13 and TIM3, a checkpoint inhibitor that was found to be upregulated in leukemic stem cells, led to improved elimination of AML. Furthermore, determination of general cancer-specific targets would alleviate the current need to target different antigens for different types of cancer. For example, designing CARs to target cancer-specific post-translational modifications such as Tn-glycosylated podoplanin (Tn-PDPN) (128) would be expected to result in fewer off-cancer effects as the 237Ab-derived 237 CAR T cells target Tn-PDPN, which is not present on normal tissue. As Tn glycosylation was present on all cancer cells evaluated, tumor escape is also less likely, which makes this a novel approach to improve CAR T cell efficacy (128). Along these lines, a genome-wide CRISPR-Cas9 screening method was recently used to discover a TCR that recognized the monomorphic MHC class I-related protein MR1 and T cells engineered to express this TCR killed several different types of human cancers without damaging healthy cells (129). Once verified, development of strategies such as these in allogeneic “off-the-shelf” cell sources could have great potential to exhibit anti-cancer activity against a broad spectrum of malignancies.

In summary, several possibilities to generate “off-the-shelf” anti-cancer immunotherapeutics are currently being explored. For example, control of TCR expression by genomic knockout or down-regulation via RNAi demonstrated the feasibility of generating “off-the-shelf” allogeneic CAR T cell products. However, also other allogeneic cell sources, such as NK cells and macrophages, appear to be suitable as “off-the-shelf” anti-cancer CAR cells. In addition to delivery of cell therapies, the possibility to apply viral vectors engineered for targeted *in vivo* modification of immune cells with CARs is another potent “off-the-shelf” strategy to generate CAR T and CAR NK cells. As research in these areas is rapidly progressing, we look forward to development of efficient “off-the-shelf” therapies that will be made broadly available to the many cancer patients world-wide.

## Author’s Note

Figures were created with BioRender.com.

## Author Contributions

All authors contributed to conception and writing of the manuscript.

## Conflict of Interest

The authors declare that the research was conducted in the absence of any commercial or financial relationships that could be construed as a potential conflict of interest.
